# Effect of frequency on fretting wear behavior of Ti/TiN multilayer film on depleted uranium

**DOI:** 10.1371/journal.pone.0175084

**Published:** 2017-04-06

**Authors:** Yan-Ping Wu, Zheng-Yang Li, Sheng-Fa Zhu, Lei Lu, Zhen-Bing Cai

**Affiliations:** 1 Institute of Materials, China Academy of Engineering Physics, Jiangyou, Sichuan, PR China; 2 Tribology Research Institute, Key Lab of Advanced Technologies of Materials, Southwest Jiaotong University, Chengdu, China; University of Akron, UNITED STATES

## Abstract

The Ti/TiN multi-layer film was prepared on the depleted uranium (DU) substrate by cathodic arc ion plating equipment. The character of multi-layer film was studied by SEM, XRD and AES, revealed that the surface was composed of small compact particle and the cross-section had a multi-layer structure. The fretting wear performance under different frequencies was performed by a MFT-6000 machine with a ball-on-plate configuration. The wear morphology was analyzed by white light interferometer, OM and SEM with an EDX. The result shows the Ti/TiN multi-layer film could greatly improve the fretting wear performance compared to the DU substrate. The fretting wear running and damaged behavior are strongly dependent on the film and test frequency. The fretting region of DU substrate and Ti/TiN multi-layer under low test frequency is gross slip. With the increase of test frequency, the fretting region of Ti/TiN multi-layer change from gross slip to mixed fretting, then to partial slip.

## Introduction

Three isotopes of uranium exist in the natural world, ^238^U, ^235^U, ^234^U, in the relative content of 99.275, 0.720, and 0.005%, respectively [1]. DU is produced as a nuclear waste in the uranium enrichment process, so the ^235^U content of DU was about 0.2–0.4% less than natural uranium. Thus it has less radio-active and usually used as DU ammunition and DU-armored tanks due to the greatly performance of armor-piercing strength [2]. However, the DU has a high chemical reactivity. When the DU is exposed to salty, humid and high temperature condition, it is easy to corrode. Hence, Al-based coating [3–5], Ti-based coating [4–6], Cr-based coatings [7, 8] and adding alloy element such as niobium [9], has been applied to enhance corrosion resistance of uranium.

Less investigation remains on the tribological performance of DU, especially for fretting wear. Fretting wear is the surface damage, which always occurs at many mechanical systems, when two contact surfaces are affected by amplitude oscillatory motion. When the DU ammunition is transported by heavy truck or train, the fretting wear phenomenon appears with the vibration frequency ranged from 2Hz to 100Hz and vibration amplitude in micrometer-scale under different velocity and road condition [10, 11]. Fretting wear is the main reason of crack and void formation in subsurface zones [12], which could lead to the failure of key component, cause huge economic losses. Surface modification and coating techniques is an effective method to prolong service life of key component. Coatings such as TiN, CrN, AlN, WN, DLC, and GLC have been widely used as protective coatings due to their excellent tribological properties. [13]. However, the single film on substrate materials is easy to be ruptured under high contact stress due to the difference of the elasticity modulus and hardness between the films and substrate. [14]. Multilayer systems can offer an efficient way of controlling residual stress or elasticity modulus, improving adhesion strength and enhancing toughness of coating [15]. The multilayer films could be well prepared by changing the thickness and composition of each single layer. Therefore, to meet the specific functional requirements for various conditions, Ti/TiN multilayers are prepared and the tribological property is studied. A specific thickness of Ti interlayer is required for improving the adhesion strength between the substrate and TiN film and reducing the residual stresses.

In this study, Ti/TiN multilayer film was deposited on the surface of DU by cathodic arc ion plating equipment. The structure and fretting behavior of Ti/TiN multilayer film and DU substrate was investigated. After the wear tests, the wear scars and wear volume were examined by laser confocal scanning microscopy (LCSM), scanning electron microscopy (SEM) and 3D optical microscope. The fretting wear mechanism of Ti/TiN multilayer film was discussed.

## Experimental

### Ti/TiN multi-layer deposition

**Fig 1:**
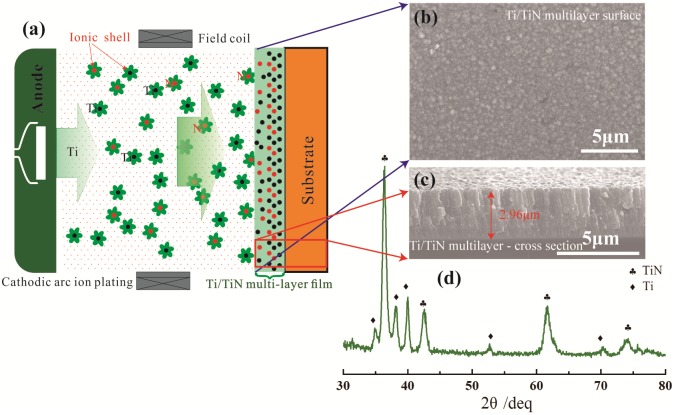
The prepared process (a), SEM surface (b), cross-section morphologies (c) of Ti/TiN multi-layer film.

The DU substrate samples used in the present study were ground with 1000 grit diamond paper and polished to a roughness of Ra = 0.5 μm, then ultrasonically cleaned in acetone and alcohol. The sputtering of Ti/TiN multi-layer film was performed using a cathodic arc ion plating equipment. The prepared process of Ti/TiN multilayer film is described by schematic in Fig 1(a). The samples were fixed on a continuously rotating planetary holder inside the vacuum chamber. Prior to deposition, the sample surface were further cleaned by argon ion bombardment for 10min to remove the native oxide layer. After the base pressure was pumped to below 5×10^−4^ Pa, the working pressure was kept at 0.3 Pa. The target was high purity Ti (99.99%). With the alternative atmosphere of pure argon and mixed argon and nitrogen controlled by mass flowmeters, the deposition procedures of Ti and TiN were repeated to prepare Ti/TiN multilayer film. For Ti film, was deposited with argon (99.999%) inlet with a flow of 40 sccm (standard-state cubic centimeter per minute). For TiN film, argon (99.999%) and nitrogen (99.999%) were introduced into the chamber with a flow of 10 and 40 sccm, respectively. The target power was 2 kW. The deposited time of Ti and TiN was 5 min and 10 min for each layer. The total deposited time was 1 h. The bias voltage was -900V×20% pulsed bias superposed by -50V DC bias.

### Film characterization

The surface and cross-section morphologies of the deposited films before and after fretting wear test were observed by scanning electron microscopy (JSM-7001F; JEOL, Japan) connected to an EDX system. The structure of Ti/TiN multi-layer film and DU substrate was analyzed by Empyrean X-ray diffraction (XRD) employing Cu kα radiation (λ = 0.15406 nm). Scanning was carried out in the grazing angle mode with an incident beam angle of 2°. AES depth profile was used to analyze the distribution of Ti and N elements. Auger electron spectroscopy (AES) instrument model was PHI650 SAM, the excitation energy was 3keV, electron beam current was 100 nA and sputtering area was ahout1mm^2^.

### Fretting tests

**Fig 2:**
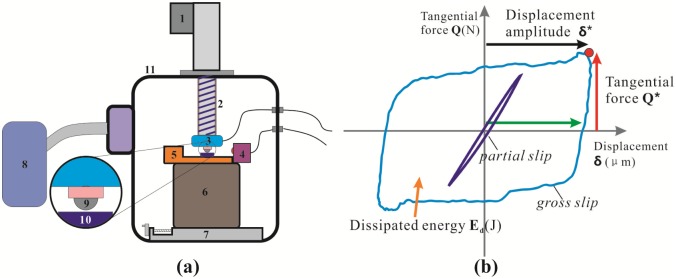
Schematic view of experimental set-up (a) and fretting loop analysis (b).

The fretting wear test was performed using a MFT-6000 machine with a ball-on-plate configuration. A schematic drawing of the fretting wear test setup and sample is shown in Fig 2(a). The normal force P was automatic applied through lead screw(2) using servo electric cylinder(1). The normal force P and tangential force Q was measured by Three-dimensional pressure sensor(3). The sliding displacement δg was induced by a piezoelectric ceramics(5), and measured by Laser copolymerization sensor(4). The base(6) was used to support the test apparatus and the screw module(7) was to adjust the position of sample. It is also a molecular pump to provide vacuum environment. In addition, the upper ball specimens(9) was GCr15 steel (wt%: 1.0 C, 1.49 Cr, 0.31 Mn, 0.26 Si, 0.009 P, 0.004 S) with a diameter of 12mm, a hardness of 766 HV and a roughness of Ra = 0.2μm. The lower sample(10) with a size of 10×10 ×30 mm was fixed in a special clamp. All the fretting tests were carried out at normal load of 20N, displacement amplitude of 20μm and frequency of 50Hz, 20Hz, 10Hz and 2Hz for 10000 fretting cycle. The test condition was conducted in room temperature dry condition with relatively humidity of 60%.

During the fretting tests, the normal force, tangential force, coefficient of friction (COF), displacement amplitude as well as number of cycles, were recorded and compared at the same time. These variables were used to draw fretting-loops for a constant normal force condition (Fig 2(b)). Diagram of a gross sliding fretting loop with the key parameters indicated: Q *, tangential force; δ*, sliding amplitude; and E_d_ (J), dissipated energy.

## Results and discussion

### As deposited film

The surface morphology and cross-section morphology of the Ti/TiN multi-layer film prepared on DU are shown in Fig 1(b) and 1(c).The SEM image shows that Ti/TiN multi-layer film is composed of many small particles and it is more compact compared to the single TiN film [16]. Because nucleation would occur at the pre-existing interfaces more easily, the existence of interfaces between Ti and TiN film increases the nucleation rate, and also leads to the suppression in the growth of columnar crystal, hence ultimately results in the compact structure. As shown in Fig 1(c), the Ti/TiN multi-layer film exhibits multilayered laminate structure, and the interface of multi-layer film can be clearly distinguished. The cross-section morphology confirms the SEM surface analysis results that the Ti/TiN multi-layer film possesses the compact microstructure without voids and cracks.

**Fig 3:**
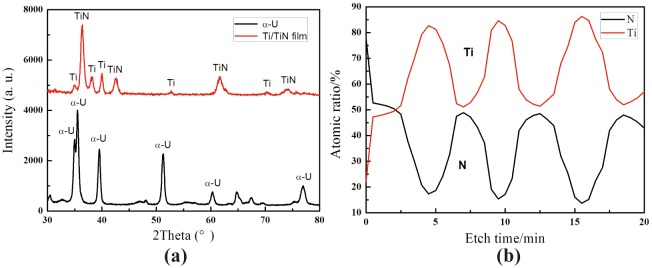
XRD patterns (a) and element depth distribution (b) of Ti/TiN multilayer film.

The XRD patterns of Ti/TiN multi-layer and DU substrate shows in Fig 3(a). The XRD pattern of the multi-layer film exhibits a preferential growth of TiN (111). The diffraction patterns are smooth and shift vertically for clarity. The α-U is clearly appeared. The typical Auger depth profiles of Ti/TiN multi-layer film on DU substrate shown in Fig 3(b) reveal that the elemental distribution of Ti and N with the film depth. On the initial surface, the high content of N elements is captured. Then the contents of N elements decrease with the increase of etch depth, while the contents of Ti elements increase. From the AES curves of Ti and N, it is noteworthy that the atomic ratio of Ti and N fluctuate periodically. When the Ti elements increase to the maximum value, the portion of N elements decrease to the minimum value, which is the layer of Ti. When the atomic ratio of Ti and N approach to 1:1, which reveal that the TiN layer appear. As a result, the change of Ti and N elements contents trend to similar sinusoid along with etch depth by AES.

### Fretting properties

**Fig 4:**
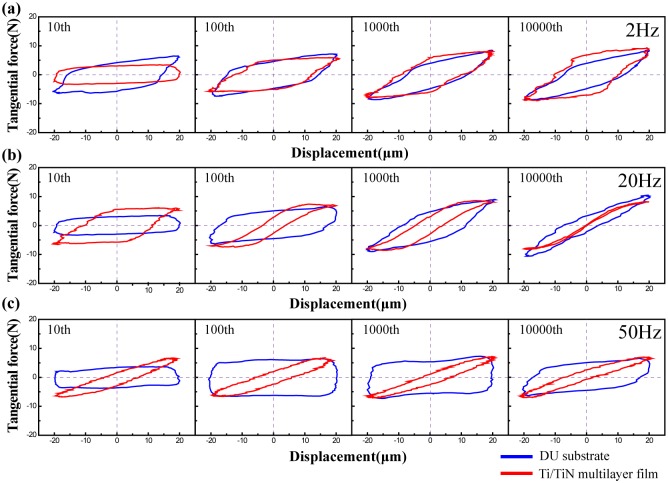
Variation of Ft–D curves as function of number of cycle under the frequency of 2Hz (a), 20Hz (b) and 50Hz (c).

As shown in Fig 4, the shapes of the F_t_-D curves are different under various frequencies. The DU sample with 2 Hz and 50 Hz and Ti/TiN film with 2 Hz is close to parallelogram, indicating that the tests are running in gross slip regime (SR). And the shape of DU and Ti/TiN film with 20 Hz is changed from parallelogram to ellipse, so it is belong to mixed fretting regime (MFR). However, the Ti/TiN film with 50 Hz is always in the shape of ellipse, thus it is divided to partial slip regime (PSR). In general, the results shows the hard coating and test frequency could affect the fretting running regime clearly, further affect the dissipated energy along with wear volume [17, 18]. Because the dissipated energy is linear correlation with wear volume.

**Fig 5:**
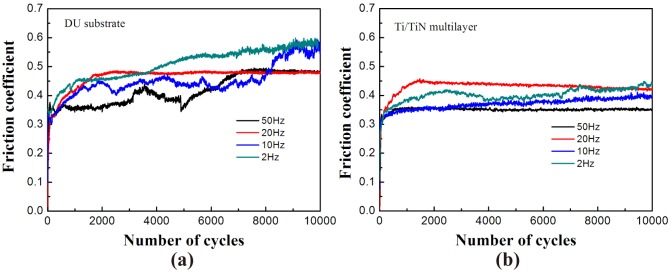
COF value of DU substrate (a) and Ti/TiN film (b) under different test frequencies.

Fig 5 shows the COF of DU substrate and Ti/TiN multi-layer film under different test frequencies. COF under the different test conditions increase rapidly after dozens of circulation to removal of oxides and the pollution layer of test surface. The COF decrease with the increased of test frequency whether DU substrate or Ti/TiN film. From the COF curve of DU substrate, the fluctuation of COF is caused by the wear debris, which does not remove out from the friction interface during the process of fretting wear. As a result, the three-body wear occur at the friction interface and lead to the fluctuation of COF. At the end, the COF under different condition become smooth and flat. On the other hand, the fluctuation of COF is due to the difference of surface roughness, which leads to the joggle of the contact interface. During the process of fretting wear, the COF become stable when the contact interface become smooth caused by wear. However, the Ti/TiN film is smoother, because the film could decrease the wear damage and wear debris [19]. Moreover, it could be the reason of the fretting regime change from SR to the MFR and PSR. Meanwhile, the COF of Ti/TiN film is lower than that of DU substrate under all test frequencies, which reveal the multi-layer film could decrease the friction.

### Morphologies of worn surfaces

**Fig 6:**
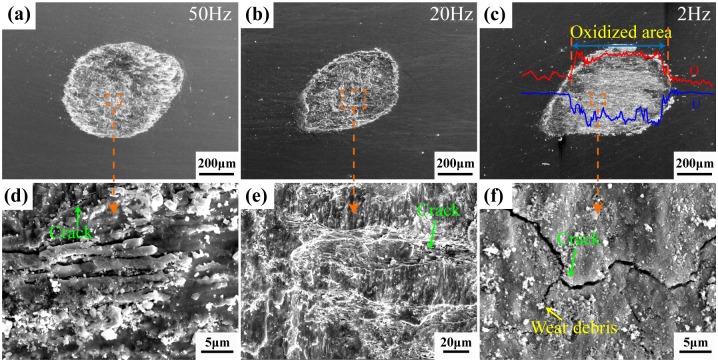
SEM images of DU substrate under the frequency of 50Hz (a), 20Hz (b) and 2Hz (c).

Fig 6 shows the SEM images of DU substrate under test frequencies of 50Hz, 20Hz, and 2Hz. From the high test frequency wear morphology of 50Hz and 20Hz, it is rough. And it has a lot of crack and intense plastic deformation, which indicate that the wear mechanism is adhesive wear due to high tangential energy and slip speed [20]. However, the wear track of 2Hz is surrounded by an accumulation of loose debris, with pockets of debris trapped within the contact region. Additionally, some cracks and delamination occurs on the wear scar, so the wear mechanism is abrasive wear [21]. Meanwhile, the tribochemical reaction is easy to occur at the friction interface due to the high chemical reactivity of DU and long-time contact with air. EDX results show the wear scar has high oxygen element content than unworn region, which demonstrate that the wear mechanism also conclud the oxidative wear.

**Fig 7:**
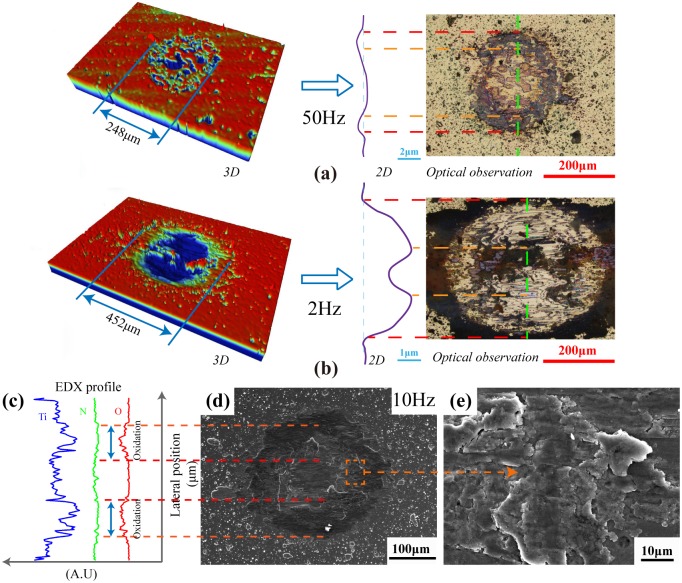
Wear morphology of Ti/TiN multilayer film after the test frequency of 50Hz (a), 2Hz (b) and 10Hz (c).

Fig 7(a) and 7(b) shows the 3D surface wear morphology of Ti/TiN multi-layer film after the test frequency of 50Hz and 2Hz, respectively. The 3D surface profile shows the surface damage of 2Hz is more serious than that of 50Hz, either width or depth. Compared with the test condition of 50Hz, wear depth increase from 0.5μm to 2.3μm that of 2Hz. From the optical image of Fig 7(b), the layered structure damage is clearly emerged from the wear scar due to the different hardness and elasticity modulus of multi-layer structure. The fretting region of 2Hz test condition is belonging to SR according to the wear morphology, which with a little protuberance in the center area. This phenomenon is caused by the three body abrasive with wear debris [22]. But the fretting region of 50Hz test condition is PSR, because it just has a slight indentation on the film surface [23]. The fretting regime analysis results through wear morphology are in accordance with the analysis results of F_t_-D curve. However, the multi-layer film is not worn out under different test frequencies, because the maximum wear depth is lower than the thickness of multi-layer film and the DU substrate is not appeared at the wear scar from optical image of Fig 7(b). Fig 7(d) is the SEM morphology of multi-layer film under test frequency of 10Hz, central region of wear scar is adhesive and partial slip occur at wear scar edge [24]. Energy dispersive spectroscopy (EDS) analysis was carried out to estimate the chemical changes induced by friction. Results show the central region of wear scar has a lower oxygen contents and edge region of wear scar has a higher oxygen contents due to the tribochemistry behavior.

**Fig 8:**
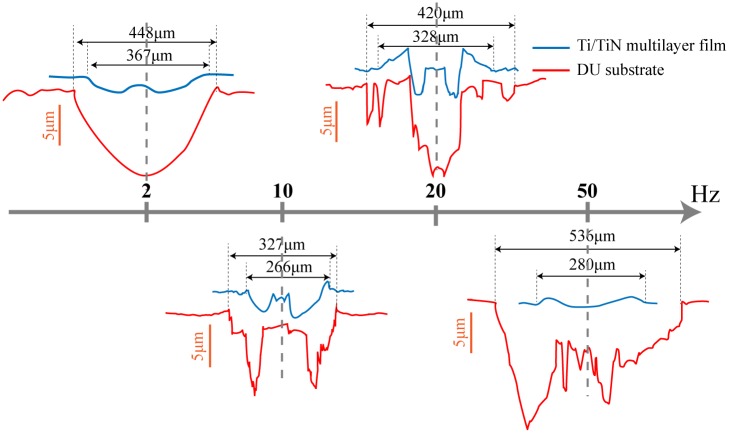
The 2D profiles of DU and Ti/TiN multilayer film under different test frequencies.

Fig 8 shows the 2D profile of DU substrate and Ti/TiN multi-layer film under all test conditions. The wear width and depth of multi-layer film sample is lower than the substrate sample due to the high hardness of Ti/TiN film, so the multi-layer film could provide protection and decrease the friction and wear effectively. The profile of DU and Ti/TiN film in 10Hz and 20Hz is accord with the profile of MFR [24]. When the test condition of DU under 2Hz and 50Hz, the wear morphology is belong to SR. As a result, the cross-sectional area of substrate sample in 2Hz and 50Hz is larger than 10Hz and 20Hz.

### Wear volume analyze

**Fig 9:**
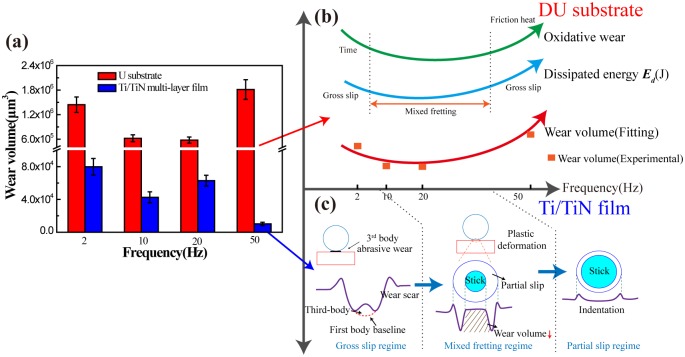
The wear volume of DU substrate and Ti/TiN multi-layer film (a), and the schematic diagram to explain the wear volume of DU (b) and Ti/TiN multi-layer film (c).

Fig 9(a) shows the wear volume of DU substrate and Ti/TiN multi-layer film under different test condition. The wear volume of Ti/TiN multi-layer film is two orders of magnitude lower than that of DU substrate. AS shown in Fig 9(b), it is a schematic diagram to explain the wear volume of DU. First the oxidative wear and dissipated energy is the main reason to influence the wear volume. Because the dissipated energy is linear correlation with the wear volume [22, 25].The oxidative wear is severe in the low and high frequency. Low frequency is corresponding to the contact time with air, while high frequency is due to the friction heat during the test. The tendency of oxidative wear is firstly decrease and then increase. Meanwhile, the dissipated energy is the same tendency with oxidative wear according to the F_t_-D curve. The test frequency of 10Hz and 20Hz is MFR, while the test frequency of 2Hz and 50Hz is SR. So the fitting of wear volume through oxidative wear and dissipated energy is just like the shape of two main wear mechanisms. The experimental of wear volume is equal proportion place on the schematic diagram. The fitting is closed to the experimental date, it also firstly decrease and then increase. From Fig 9(c), we just analyze the wear volume caused by the dissipated energy or the fretting regime of Ti/TiN film. Because the oxidative wear is not the main wear mechanism, so we do not consider it. When the test condition is 2Hz, it is belonging to SR, and the wear scar is a little protuberance in the center area, just like the Fig 9(c) of SR wear scar. It is caused by the three body abrasive with wear debris [22]. Hence, it has a maximum wear volume. While the test condition is 10Hz and 20Hz, it is MFR. The center area of wear scar is stick, and partial slip occurs at wear scar edge. Therefore, the central area of wear scar is not damaged, the wear volume decrease. But the fretting region of 50Hz is partial slip, because it just has a slight indentation on the film surface [23]. It could be the reason of elastic deformation between the different layers of multilayer to resist the relative slip of contact surfaces under the high frequency [26]. Hence, the COF of the Ti/TiN multi-layer in 50Hz is minimum and stable.

## Conclusion

The Ti/TiN multi-layer film was prepared on the DU substrate, the character of multi-layer film was conduct by SEM, XRD and AES, revealed that the Ti/TiN multi-layer surface was composed of many small particles and compact. The fretting wear performance was performed under different frequencies. The observation and analysis led to the following conclusions:

1. The Ti/TiN multi-layer film could greatly decrease the friction and wear compared to the DU substrate, almost two orders of magnitude of wear volume.
2. The fretting region of DU substrate and Ti/TiN multi-layer under low test frequency is gross slip. While with the increase of test frequency, the fretting region of Ti/TiN multi-layer change from gross slip to mixed fretting, then to partial slip.
3. The wear mechanism of DU substrate under high test frequency is adhesive wear. However, under low test frequency is exfoliation, abrasive and oxidative wear.
4. It is effective to use the Ti/TiN multi-layer to reduce the friction and wear, especially for high vibration frequency.

## Supporting information

S1 File Supporting information file. (ZIP)
