# Supplementary figures and images for: Effect of frequency on fretting wear behavior of Ti/TiN multilayer film on depleted uranium
Source: PLoS One. 2017 Apr 6;12(4):e0175084. doi: 10.1371/journal.pone.0175084 (PMC5383101; doi:10.1371/journal.pone.0175084)

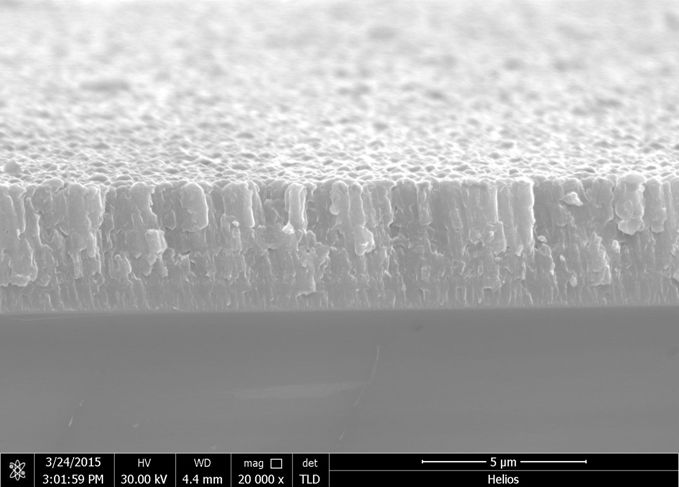

Supplement: S1 File — (ZIP) [file pone.0175084.s001.zip › Supporting Information file/Fig 1/Cross section.jpg]

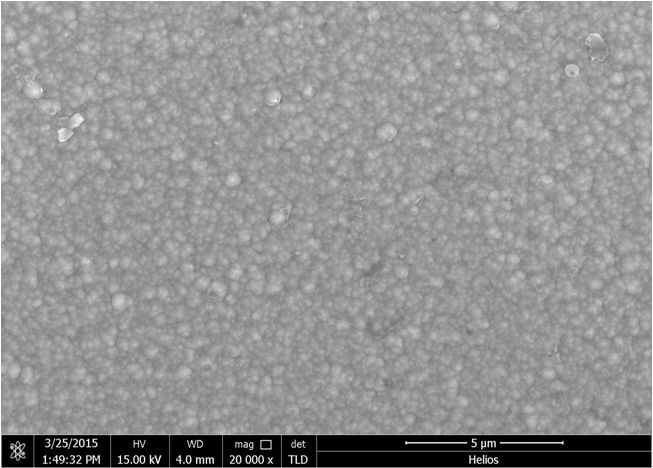

Supplement: S1 File — (ZIP) [file pone.0175084.s001.zip › Supporting Information file/Fig 1/surface.jpg]

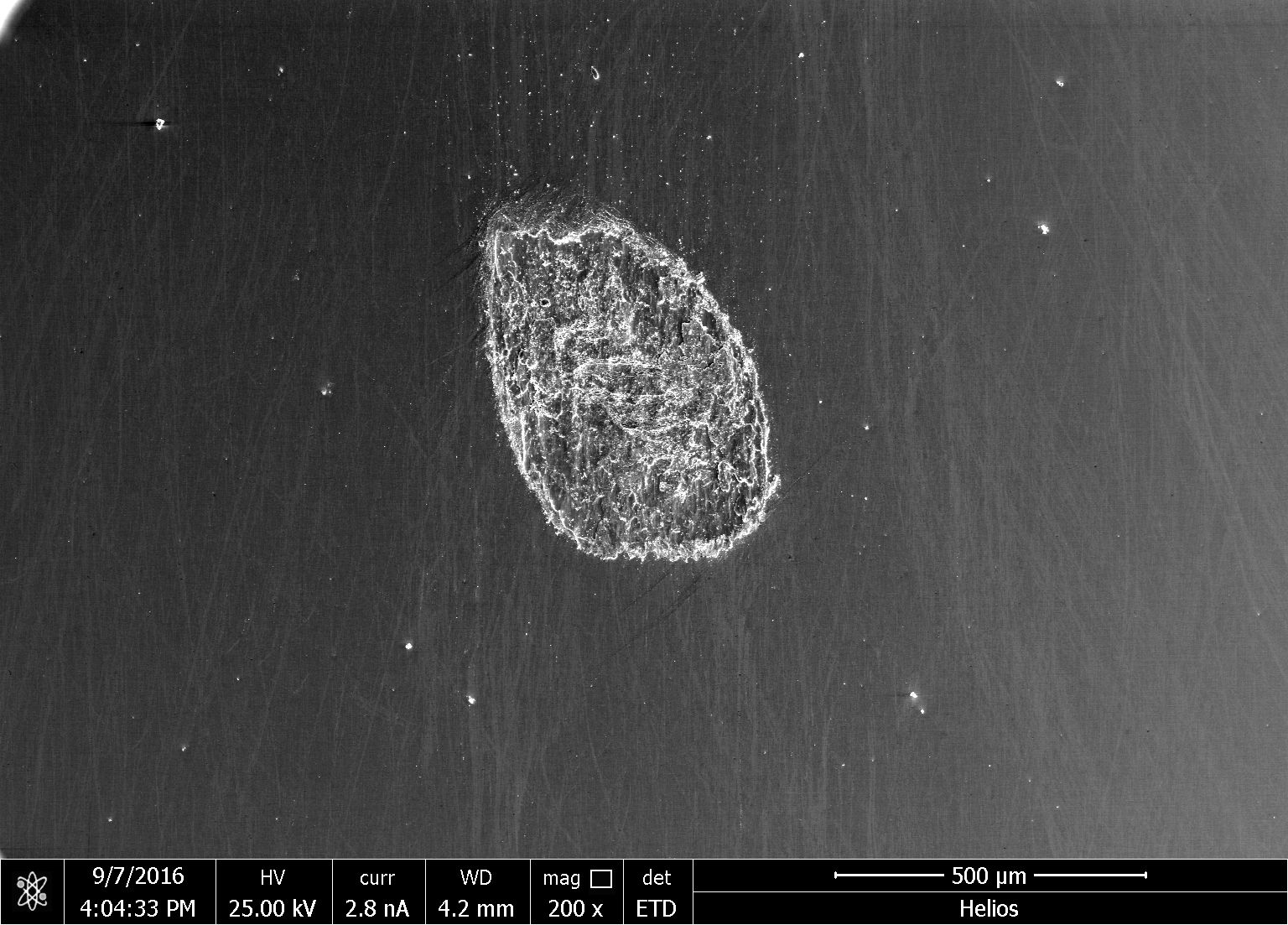

Supplement: S1 File — (ZIP) [file pone.0175084.s001.zip › Supporting Information file/Fig 6/20hz-1.jpg]

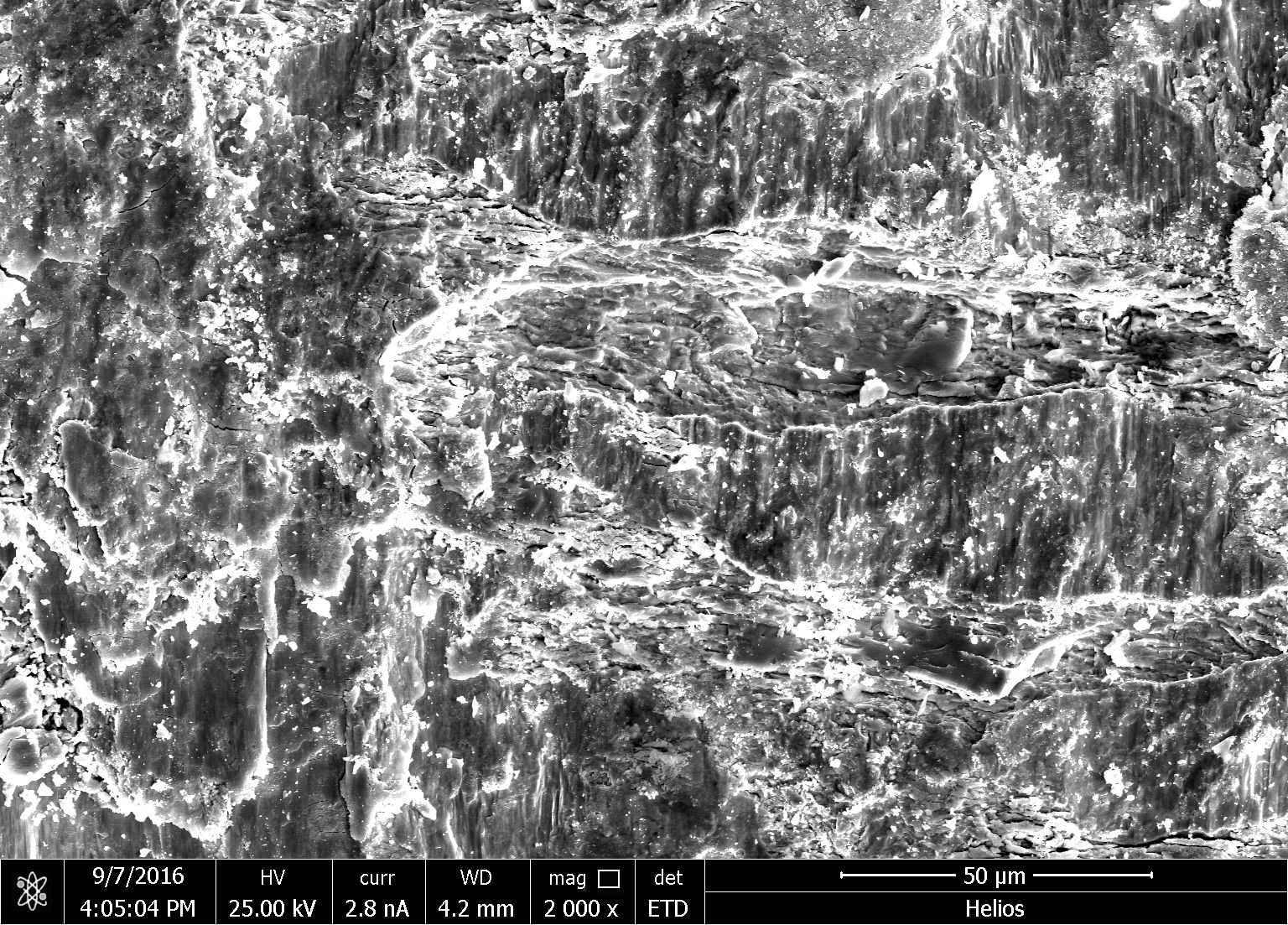

Supplement: S1 File — (ZIP) [file pone.0175084.s001.zip › Supporting Information file/Fig 6/20hz-2.jpg]

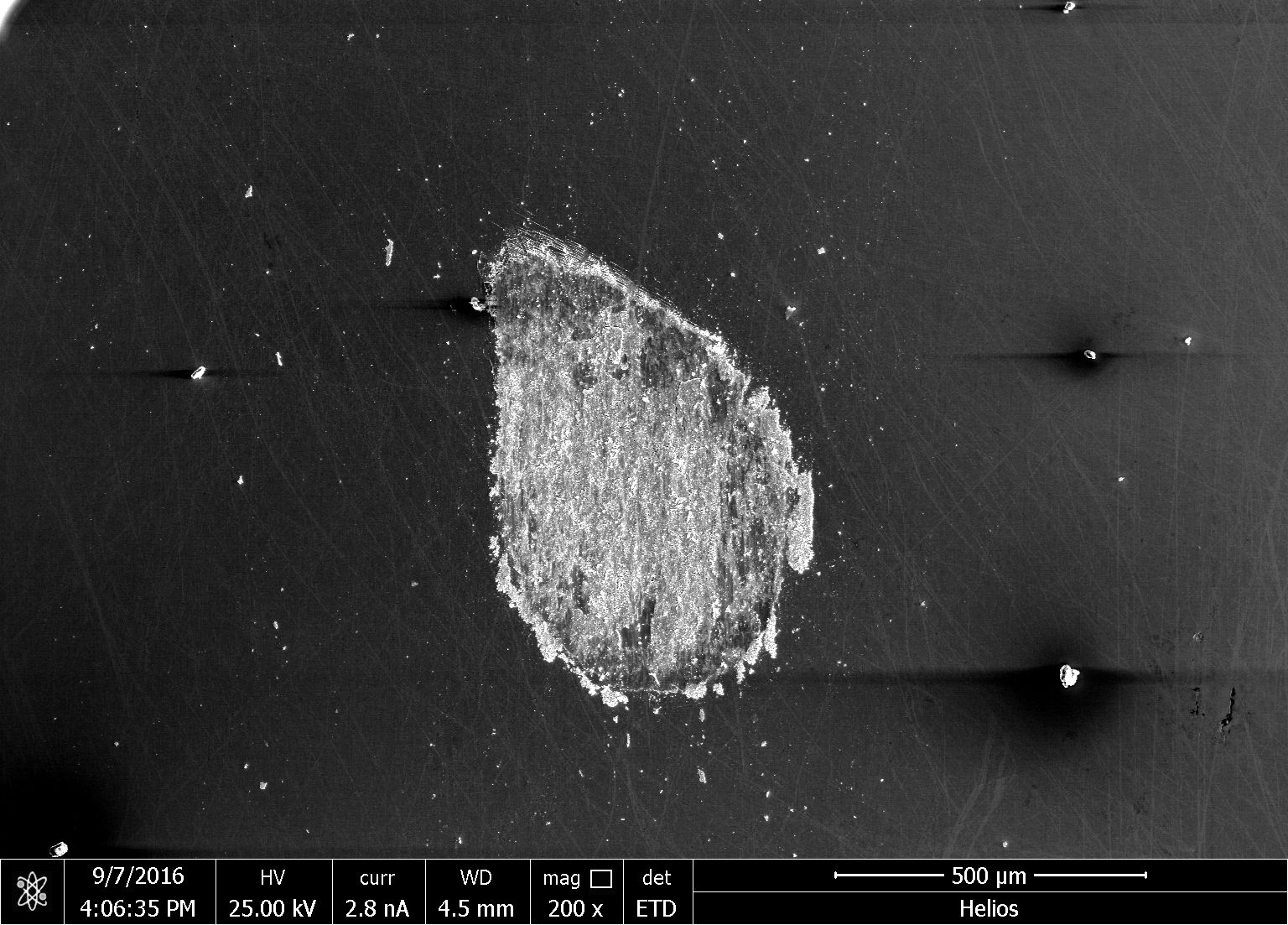

Supplement: S1 File — (ZIP) [file pone.0175084.s001.zip › Supporting Information file/Fig 6/2hz-1.jpg]

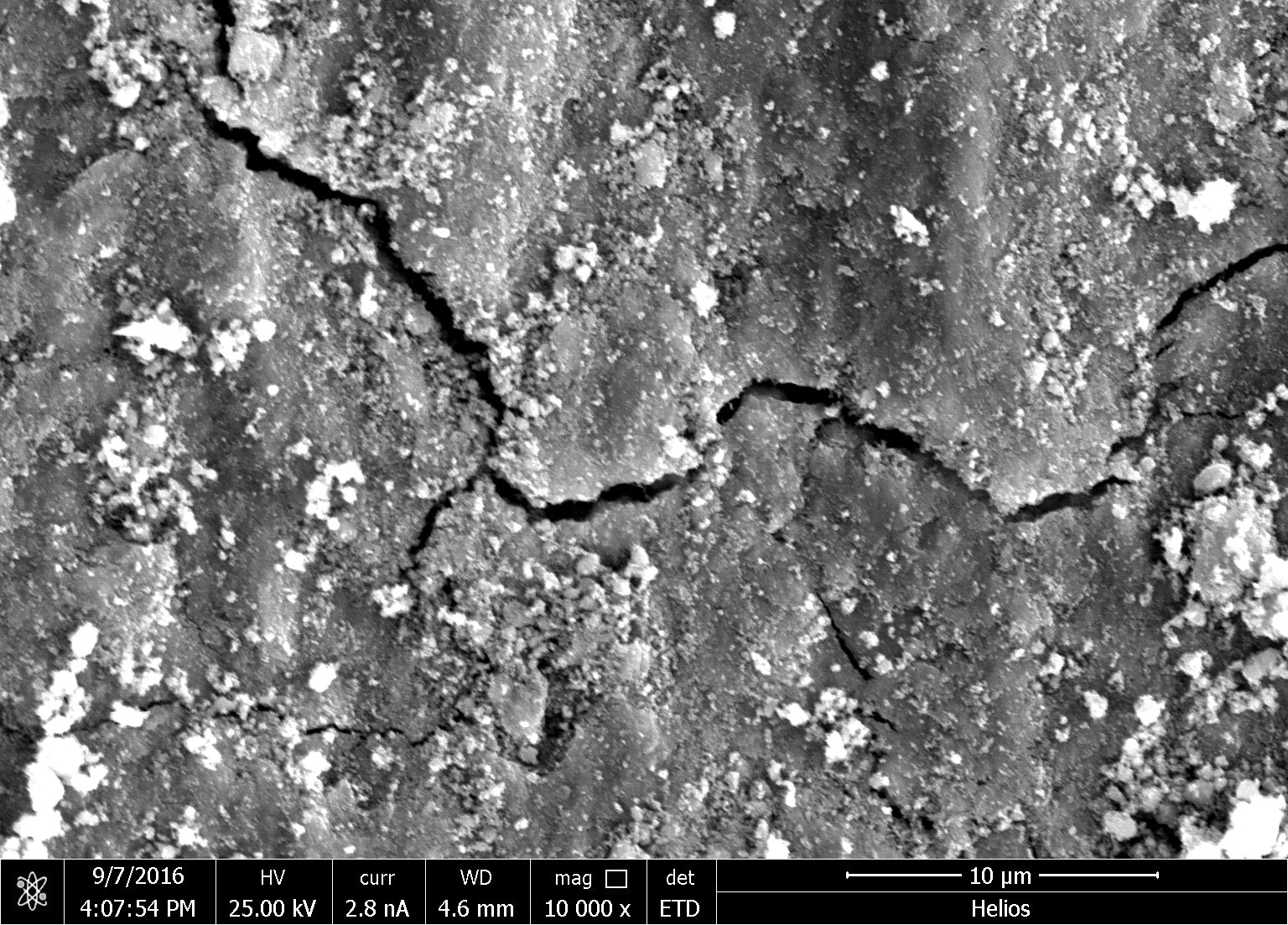

Supplement: S1 File — (ZIP) [file pone.0175084.s001.zip › Supporting Information file/Fig 6/2hz-3.jpg]

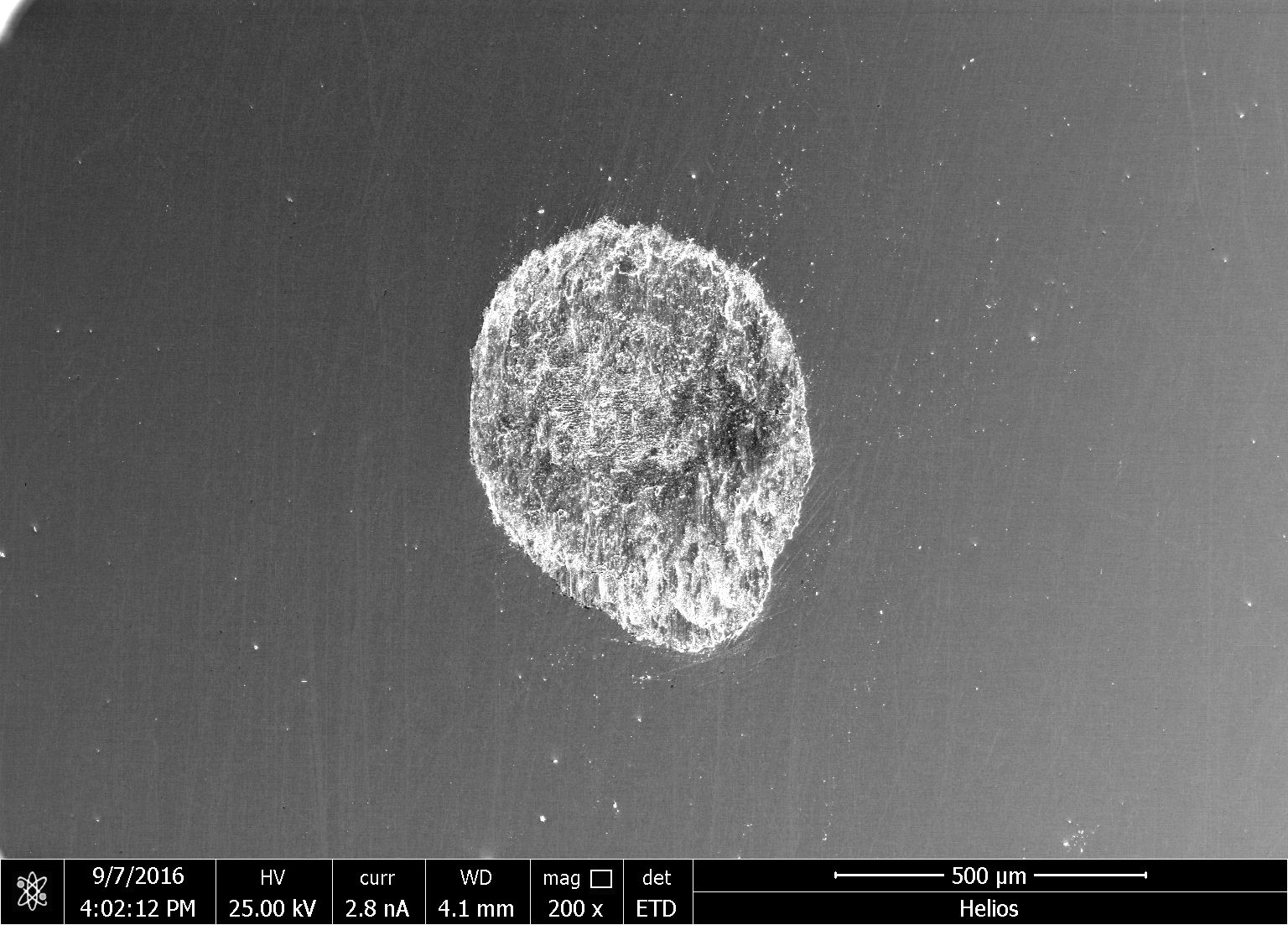

Supplement: S1 File — (ZIP) [file pone.0175084.s001.zip › Supporting Information file/Fig 6/50hz-1.jpg]

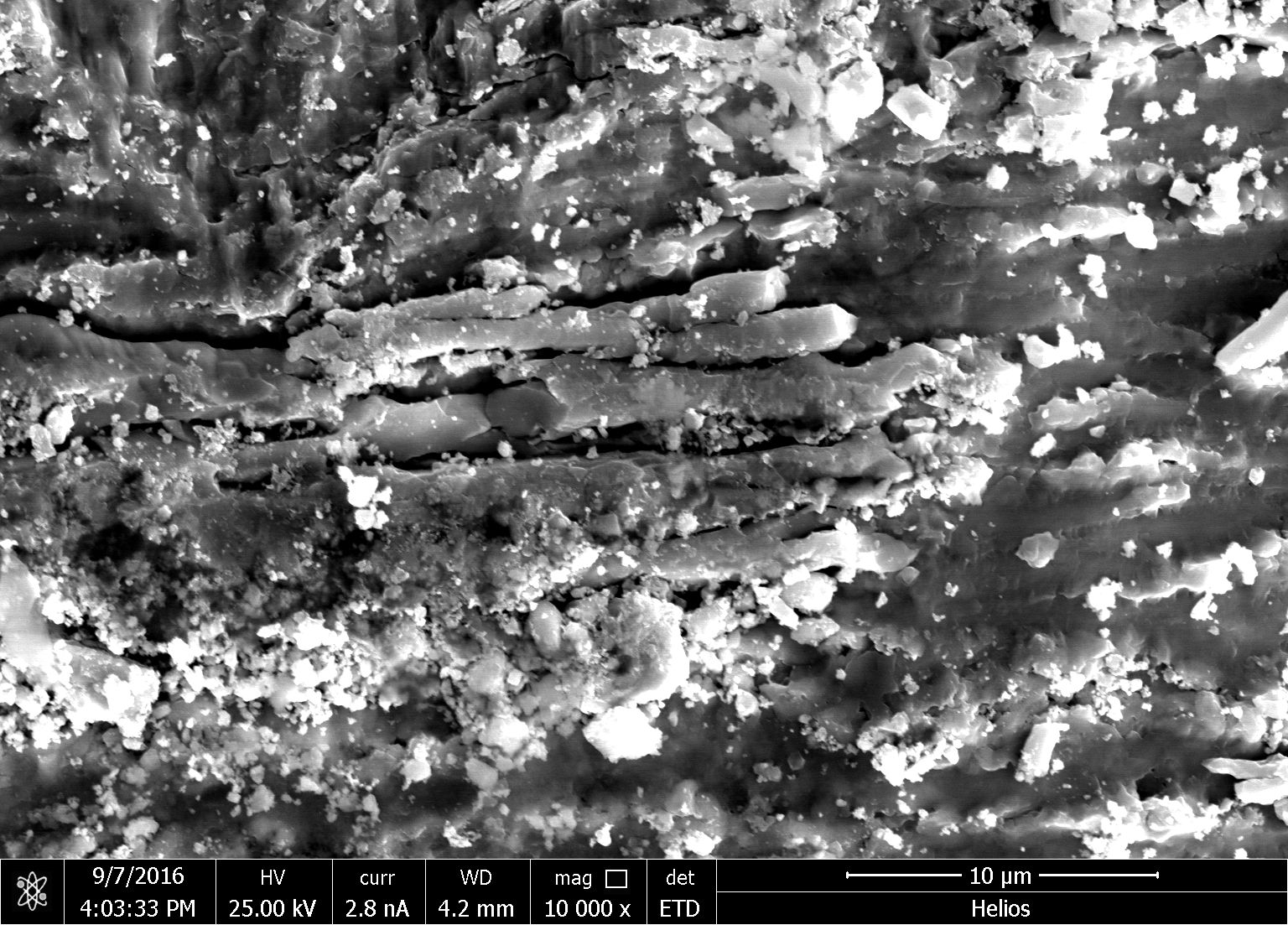

Supplement: S1 File — (ZIP) [file pone.0175084.s001.zip › Supporting Information file/Fig 6/50hz-2.jpg]

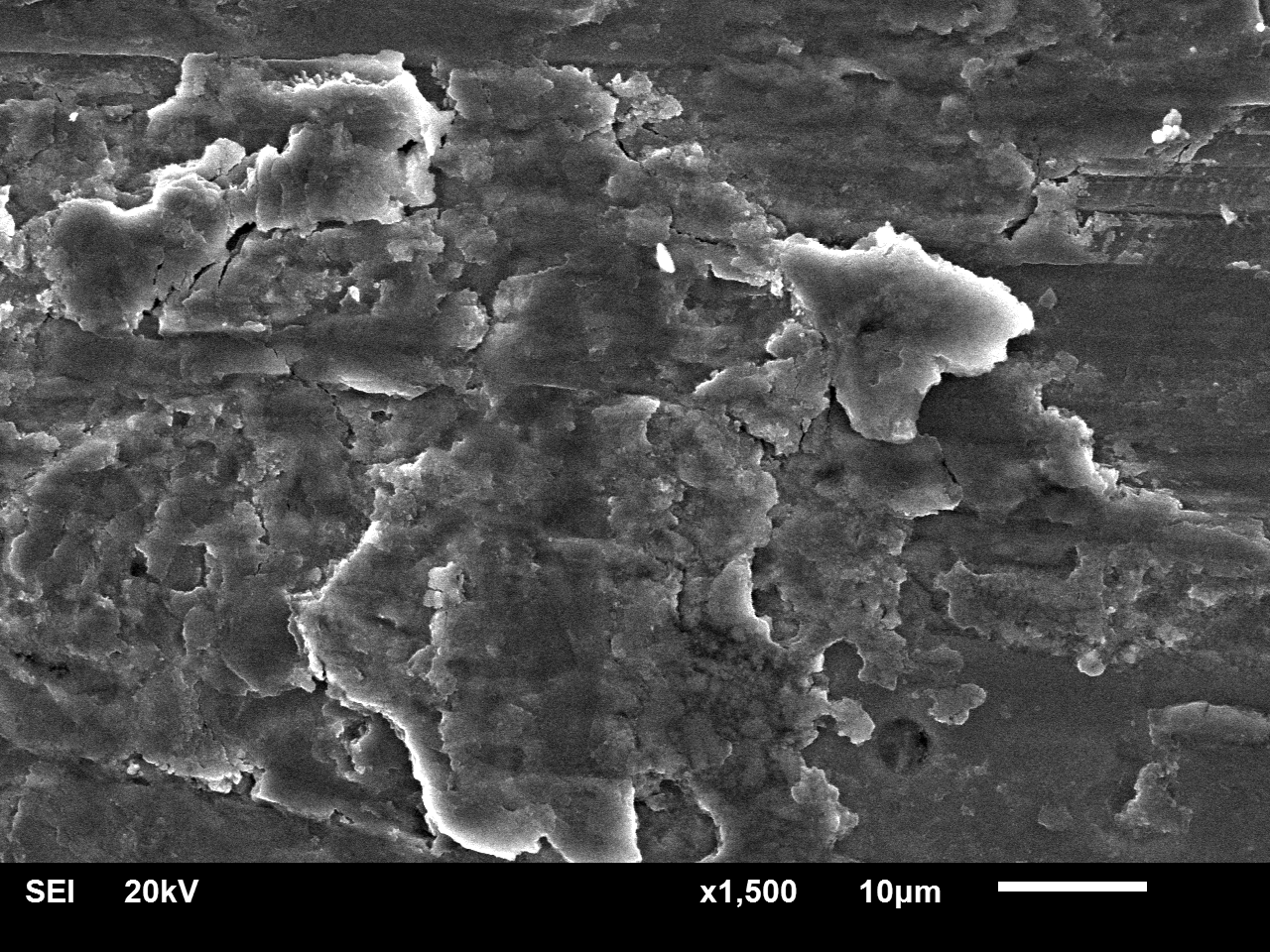

Supplement: S1 File — (ZIP) [file pone.0175084.s001.zip › Supporting Information file/Fig 7/10Hz-1500▒╢.bmp]

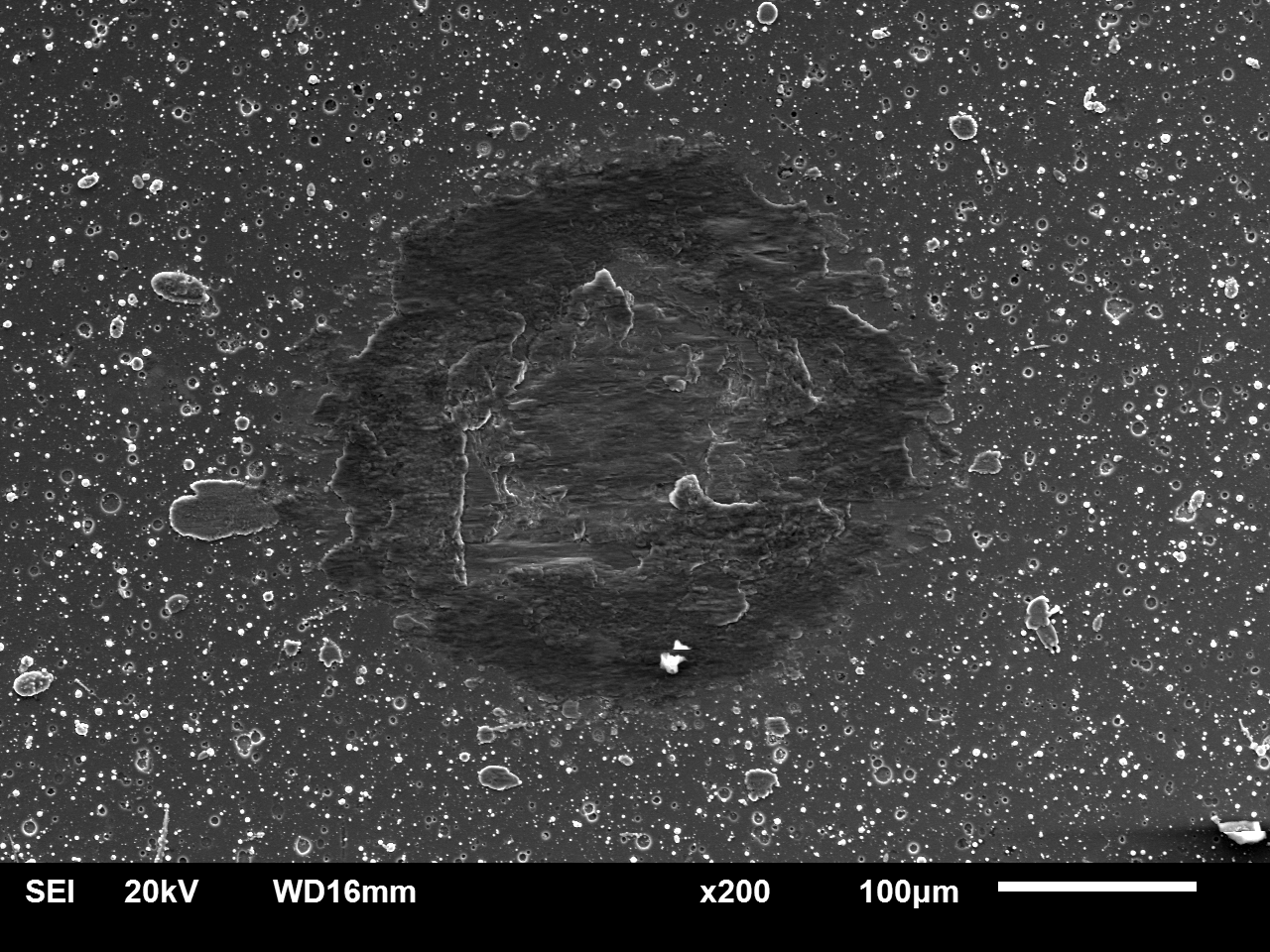

Supplement: S1 File — (ZIP) [file pone.0175084.s001.zip › Supporting Information file/Fig 7/10Hz.bmp]

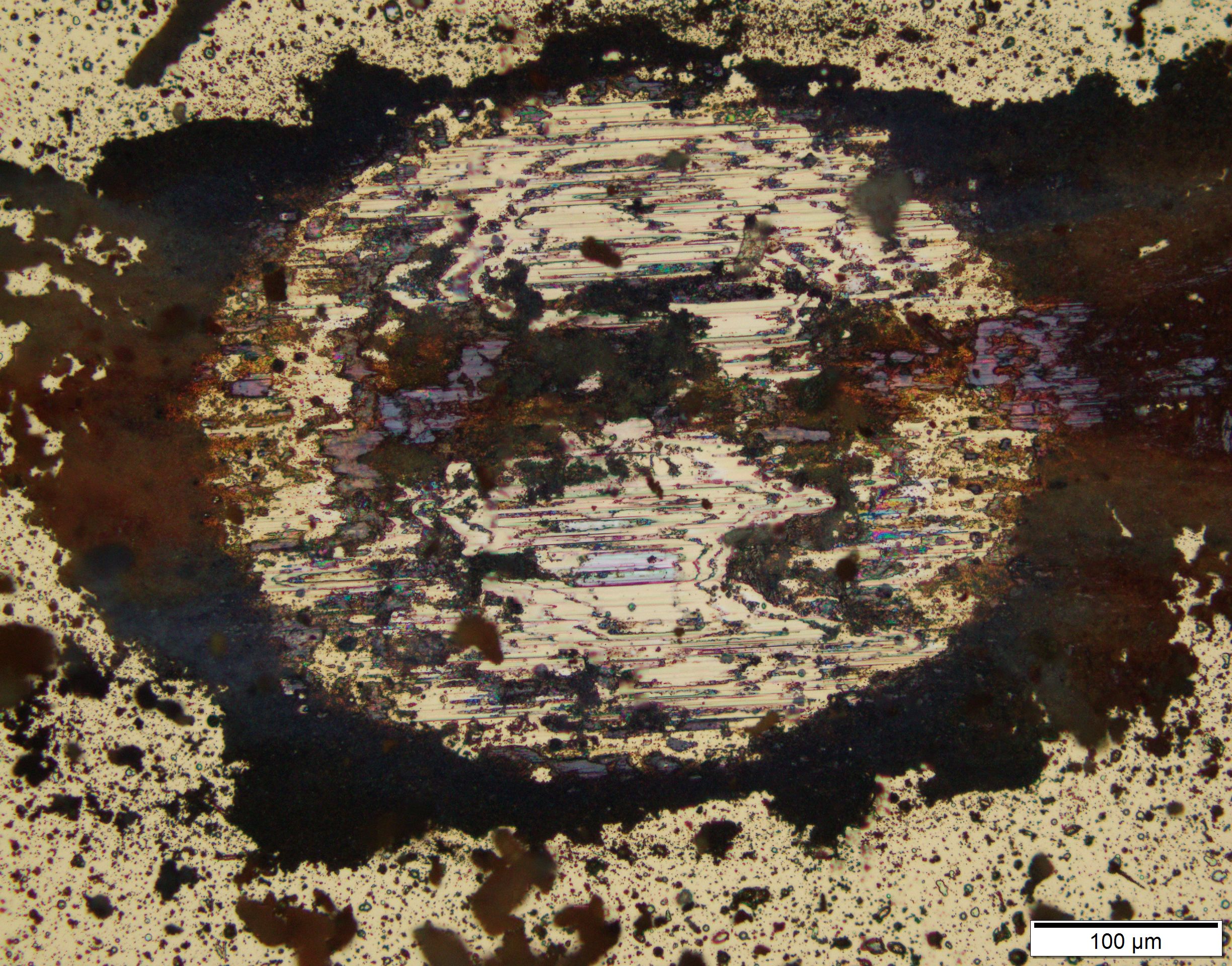

Supplement: S1 File — (ZIP) [file pone.0175084.s001.zip › Supporting Information file/Fig 7/20N-20um-2Hz.JPG]

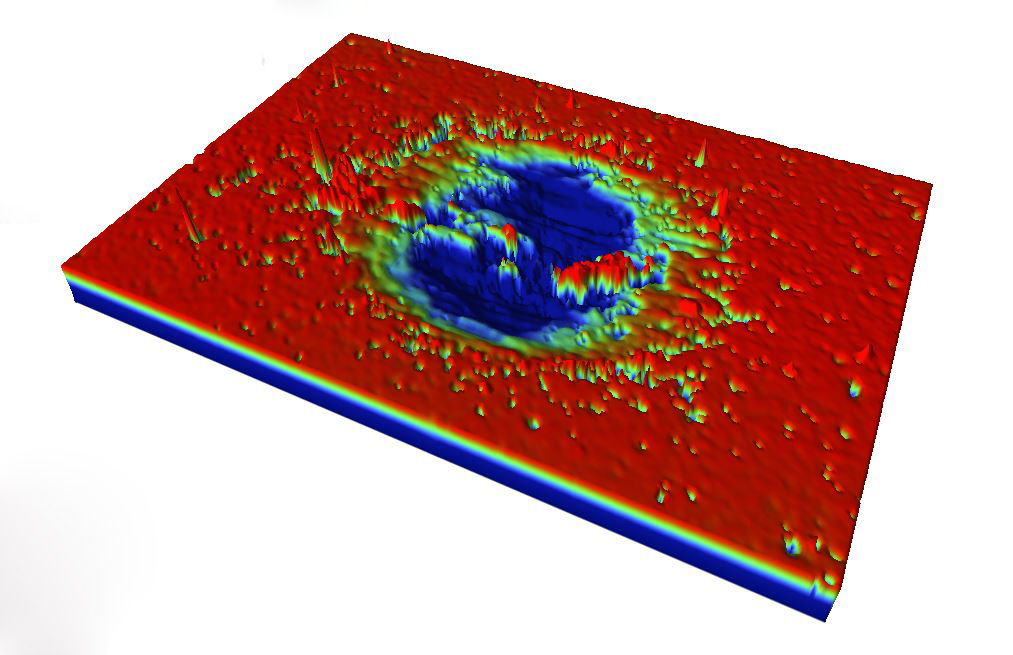

Supplement: S1 File — (ZIP) [file pone.0175084.s001.zip › Supporting Information file/Fig 7/2hz.jpg]

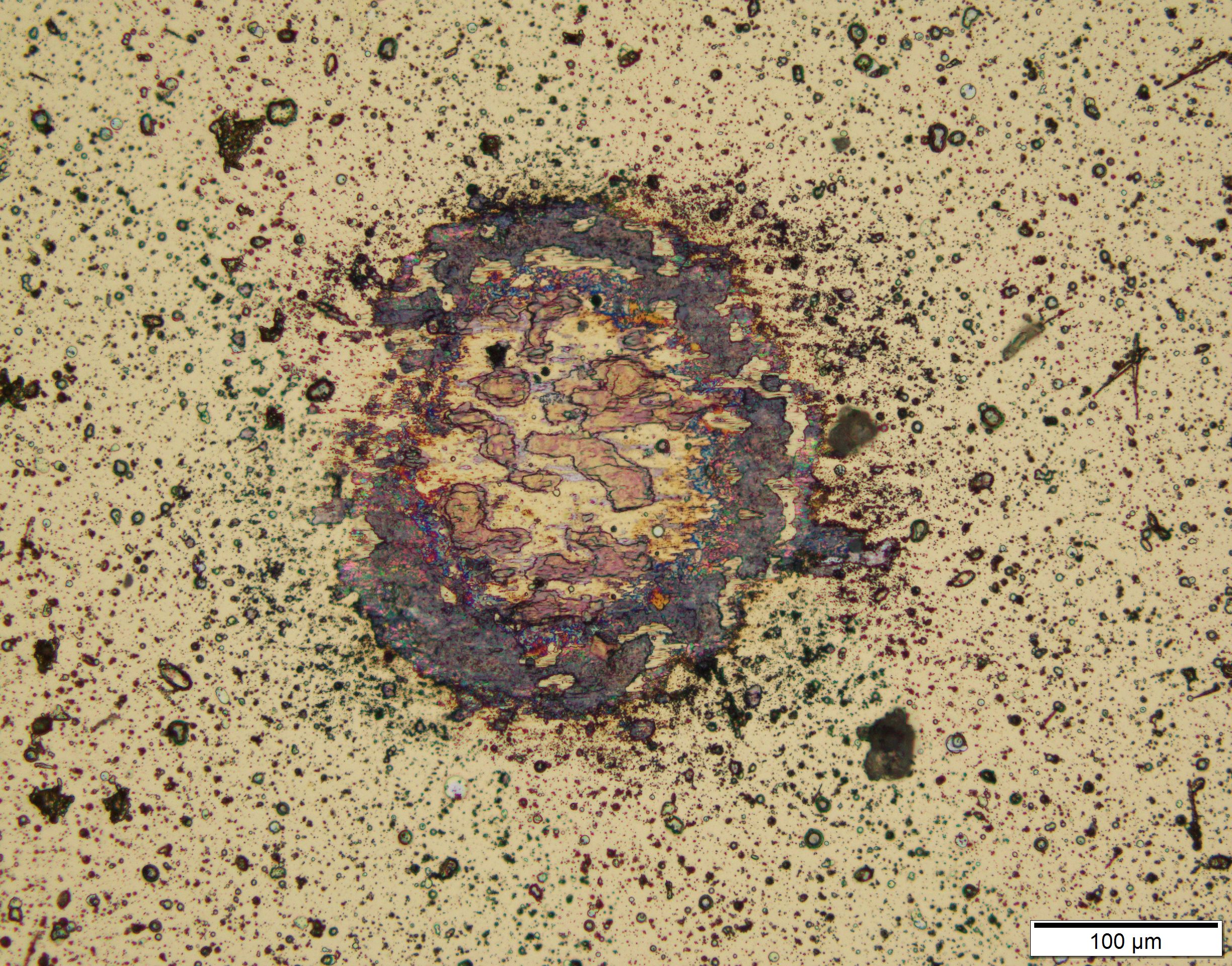

Supplement: S1 File — (ZIP) [file pone.0175084.s001.zip › Supporting Information file/Fig 7/50Hz-20um-20N.JPG]

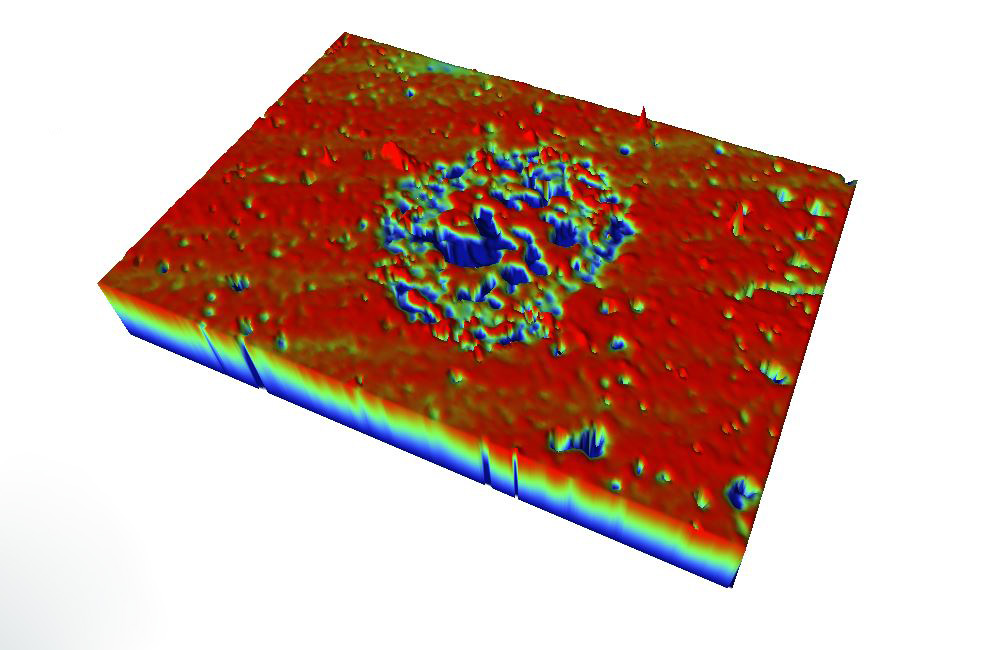

Supplement: S1 File — (ZIP) [file pone.0175084.s001.zip › Supporting Information file/Fig 7/50hz.jpg]
